# A novel small molecule inhibitor of p32 mitochondrial protein overexpressed in glioma

**DOI:** 10.1186/s12967-017-1312-7

**Published:** 2017-10-18

**Authors:** Venkata Yenugonda, Natsuko Nomura, Valentina Kouznetsova, Igor Tsigelny, Valentina Fogal, Elmar Nurmemmedov, Santosh Kesari, Ivan Babic

**Affiliations:** 1 John Wayne Cancer Institute and Pacific Neuroscience Institute at Providence Saint John’s Health Center, Santa Monica, CA USA; 2 University of California San Diego, La Jolla, CA USA

**Keywords:** Mitochondrial p32, C1qBP, Glioma, Metabolism, Pharmacophore modeling

## Abstract

**Background:**

The mitochondrial protein p32 is a validated therapeutic target of cancer overexpressed in glioma. Therapeutic targeting of p32 with monoclonal antibody or p32-binding LyP-1 tumor-homing peptide can limit tumor growth. However, these agents do not specifically target mitochondrial-localized p32 and would not readily cross the blood–brain barrier to target p32-overexpressing gliomas. Identifying small molecule inhibitors of p32 overexpressed in cancer is a more rational therapeutic strategy. Thus, in this study we employed a pharmacophore modeling strategy to identify small molecules that could bind and inhibit mitochondrial p32.

**Methods:**

A pharmacophore model of C1q and LyP-1 peptide association with p32 was used to screen a virtual compound library. A primary screening assay for inhibitors of p32 was developed to identify compounds that could rescue p32-dependent glutamine-addicted glioma cells from glutamine withdrawal. Inhibitors from this screen were analyzed for direct binding to p32 by fluorescence polarization assay and protein thermal shift. Affect of the p32 inhibitor on glioma cell proliferation was assessed by Alamar Blue assay, and affect on metabolism was examined by measuring lactate secretion.

**Results:**

Identification of a hit compound (M36) validates the pharmacophore model. M36 binds directly to p32 and inhibits LyP-1 tumor homing peptide association with p32 in vitro. M36 effectively inhibits the growth of p32 overexpressing glioma cells, and sensitizes the cells to glucose depletion.

**Conclusions:**

This study demonstrates a novel screening strategy to identify potential inhibitors of mitochondrial p32 protein overexpressed in glioma. High throughput screening employing this strategy has potential to identify highly selective, potent, brain-penetrant small molecules amenable for further drug development.

## Background

We previously reported that the gene encoding the protein p32 (*C1qBP*) is a direct transcriptional target of the *cMyc* oncogene [1]. It is overexpressed in glioma and its expression correlates with malignancy grade [1]. Higher levels of p32 expression have been reported for numerous types of cancers including breast, endometrial, ovarian, prostate, melanoma, and colon, suggesting a potential role in tumorigenesis [2–6]. P32 is predominantly localized to the mitochondria where it has a functional role in maintaining oxidative phosphorylation (OxPhos) [1, 7–10]. Loss of p32 switches metabolism from mitochondrial OxPhos to aerobic glycolysis. The functional role of p32 in the mitochondria is not completely understood. The reported crystal structure of p32 revealed it forms a homo trimer, and it was suggested that p32 might function as a regulator of Ca^2+^ ion concentration in the mitochondrial matrix, and mitochondrial Ca^2+^ levels are strongly coupled with OxPhos [11]. Mitochondrial p32 regulates cancer cell metabolism and has a role in mediating Myc-induced glutamine addiction [1, 7, 8]. Loss of p32 in glutamine addicted glioma cells induced resistance to glutamine deprivation and sensitivity to glucose withdrawal [1]. P32 appears to be critical for tumor progression. Genetic knockdown of p32 significantly reduced tumor formation in vivo [1, 8]. Thus p32 is a potential important therapeutic cancer target.

Although p32 is considered a mitochondrial matrix protein it is also localized to the cell surface and may have multiple functions [12–17]. In vivo targeting of cell surface expressed p32 with a monoclonal antibody inhibits tumor growth, thus demonstrating p32 provides a growth advantage to tumor cells [18, 19]. The exact mechanism of cell growth inhibition by anti-p32 antibodies is not completely understood, but may be due to the inhibition of cell surface p32, which prevents lamellipodia formation and cell migration [18]. A tumor-homing peptide, LyP-1 (CGNKRTRGC), targets the cell surface localized p32 [12, 20–22]. LyP-1 has been used to deliver nanoparticles to breast tumors overexpressing p32, and has tumor growth inhibition effects in vivo [20]. However, a limitation of both anti-p32 antibody and the LyP-1 peptide is that only the subset of p32 that is exposed on the cell surface can be targeted. P32 is predominantly localized to the mitochondria, and it would be advantageous for p32-directed therapeutics to inhibit the protein in all subcellular compartments. In addition, both antibody and peptide are too large to cross the blood–brain barrier, and may not be useful for targeting p32 overexpressed in brain cancers. Identifying a small molecule inhibitor of p32 represents a better therapeutic strategy. Since p32 is a validated cancer target, we expect a small molecule inhibitor of p32 to be an effective therapeutic for glioma and other types of cancers overexpressing p32—e.g., melanoma, breast, colon, and prostate cancers [23].

Here we describe and validate a pharmacophore modeling approach to identify potential small molecule inhibitors of p32. Molecular configurations of LyP-1 peptide binding to p32, and C1q binding p32, are shown to have similarities and can be superimposed. A consensus 3D pharmacophore model was developed based on common pharmacophore-centers features of these two complexes. This model was used to screen for compounds that would fit such pharmacophore. A primary screening assay employing a glutamine-addicted glioma cell line identifies a hit compound (M36). The potential p32 inhibitor binds directly to p32 and inhibits p32 association with LyP-1. We further demonstrate M36 can phenocopy p32 genetic knockdown, and can inhibit the growth of glioma cells and patient-derived glioma stem cells in culture.

## Methods

### Homology and pharmacophore modeling

#### Modeling protein loops of p32 protein

P32 crystal structure (PDB ID: 1p32) consists of the three similar chains. Each chain is encoded with 282 residues. NH_2_ terminus started at residue 74. Residues missing: In the chain A—141–161 (PTFDGEEEPSQGQKVEEQEPE), 191–196 (DEVGQE); in the chain B—139–163 (IPPTFDGEEEPSQGQKVEEQEPELT), 190–201 (PEDEVGQEDEAES); in the chain C—140–160 PPTFDGEEEPSQGQKVEEQEP), 190–201 (PEDEVGQEDEAES). The Loop Modeler module of MOE 2015 package (CCG, Montreal, Canada) was used to replace missing residues.

#### Docking of C1q with p32

Docking of C1q (PDB ID: 1pk6) with p32 (PDB ID: 1p32) was conducted using the Docking module of InsightII program (Accelrys, San Diego, Calif.). During docking, we used experimental data elucidating the binding region of C1q (Arg158, 162, 163; Ser156; and Val161 to p32 (Asp77, 79, 84, 88 and Glu89, 92, 93) [13, 24]. Then a binding fragment of C1q (residues 152–164: SIVSSSRGQVRRS) was separated.

#### Peptide modeling of LyP-1

Cyclic peptide LyP-1 (CGNKRTRGC) was constructed using Protein Builder module of InsightII package. Then sulfur bonds (CYS1–CYS9) were fixed and restrained molecular dynamics was performed taking in consideration the sulfur bond between Cys1 and Cys9.

#### Superposition of LyP-1 with binding fragment of C1q

Superposition of the cyclic peptide LyP-1 with binding fragment of C1q was performed using Align/Superpose module of Sequence Editor of MOE 2015.

#### Pharmacophore development and database screening

A six-feature pharmacophore was developed with the MOE 2015 Pharmacophore Query Editor. Pharmacophore centers were based on superimposed C1q–LyP-1 residues.

The Open NCI Database (http://cactvs.nci.nih.gov/download/nci/) release 4, containing 3D structures of over 260,000 compounds was virtually screened with Pharmacophore Search module of MOE 2015 for compounds that would fit the pharmacophore.

#### Similarity clustering of compounds

For similarity clustering, we used Fingerprint Clusters module of MOE. We used the GpiDAPH3 fingerprint and similarity-overlapping parameter *SO* = 40%. For more accurate clustering *SO* = 45% and *SO* = 53% were chosen.

### Cell lines and reagents

SF188, U373, and UW426 cell lines were cultured in DMEM with 4.5 g/L glucose and l-Glutamine without sodium pyruvate (Corning Cellgro, cat# 10-017-CV) supplemented with 10% fetal bovine serum (FBS) (HyClone), 1 × penicillin streptomycin solution (Corning) at 37 °C/5% CO_2_. GBM8 is a patient derived glioma stem cell line and was obtained and cultured as neurospheres as previously described [25, 26]. Sphere cultures were passaged by dissociation, washing and resuspension in neural stem cell culture medium (NeuroCult™ NS-A Proliferation kit #05751, Stemcell Technologies), according to the manufacturer’s instructions. Institutional Review Boards of the UC San Diego Human Research Protections Program reviewed and approved this project (IRB # 100936), in accordance with the requirements of the Code of Federal Regulations on the Protection of Human Subjects. The IRB granted a waiver of informed consent for the recruitment component of this project.

For crystal violet staining cells were grown in 24 wells and incubated with indicated concentrations of inhibitor for 72 h. Cells were washed twice with PBS, incubated at room temperature with 0.2% crystal violet in 2% ethanol 10 min, washed gently with water several times then imaged.

Antibodies used for immunoblot analysis were: anti-C1QBP (D7H12) XP Rabbit mAb from Cell Signaling Technology (cat#6502), and anti-beta-Actin (13E5) Rabbit mAb from Cell Signaling Technology (cat#4970).

### Cell viability assay

GBM8 cells were seeded at 2500 cells per well in 96-well plates. The next day increasing concentrations of inhibitor was added. After 4 days neurospheres were viewed and photographed under a Nikon microscope (4 × objective). Cell viability was determined by Alamar Blue assay after 8 days (Thermo Fisher Scientific).

For cell viability in high or low glucose, glioma cells were seeded in 96-well plates (2500 cells per well). The following day cells were washed twice with PBS, and glucose free DMEM supplemented (DMEM 1X, with l-Glutamine, without d-Glucose, without sodium pyruvate) (Gibco by Life Technologies, cat#11966-025) with 1% dialyzed FBS (Gibco by Life Technologies, cat#26400-036), penicillin–streptomycin (Corning), and d-(+)-Glucose solution (SIGMA), to either 2 or 25 mM final concentration, was added. Increasing inhibitor concentrations were added and incubated with cells for 72 h. Cell viability was determined by Alamar Blue assay (Thermo Fisher Scientific).

### Protein thermal shift assay

Human recombinant p32 (OriGene) was tested in a reaction volume of 20 µL with 100 µM of p32 inhibitor. The thermal shift reaction was performed with a BioRad CFX96 real-time PCR machine, and analysis for binding induced shifts in thermal transition was performed with Precision Melt Analysis Software provided by the manufacturer (BioRad).

### Lactate assay

Glioma cells or neurospheres were plated in 96 well plates and treated with either DMSO control or p32 inhibitor at 50 µM for 4 days (SF188 cells) or 7 days (patient-derived neurospheres). Medium was collected and tested for lactate concentration using an l-Lactate assay kit (Eton Biosciences Inc.). Sytox Green nucleic acid stain (Thermo Fisher Scientific) was used to normalize for changes in cell number. Specifically, Sytox green was added to the cells at a final concentration of 5 µM and 100 µL of 0.4% Triton-X-100 solution was added (0.2% final concentration) and incubated for 30 min at room temperature in the dark. Fluorescence at 485ex/520em was measured using a fluorescence plate reader (POLARstar Omega). Relative lactate levels were obtained after normalizing by total cell number.

### Primary screening assay in glutamine free media

SF188 glioma cells were seeded at 5 × 10^4^ cells per well in a 12 well plate. The next day cells were washed twice with PBS and then glutamine free DMEM media added (DMEM 1 ×, with 4.5 g/L d-Glucose, without l-Glutamine and without sodium pyruvate) (Gibco by Life Technologies, cat#11960-044), and supplemented with 10% dialyzed FBS (Gibco by Life Technologies). After 3–4 days the media was removed and complete DMEM (with 2 mM glutamine and 10% FBS) was added to allow cells to recover for 24 h. Cell viability was determined by Alamar Blue assay (Thermo Fisher Scientific).

### Fluorescence polarization (FP) assay

5FAM labeled LyP-1 peptide: [5FAM] G [C] GNKRTRG [C] [COOH] was synthesized by ThermoFisher Scientific. The peptide was made cyclic by disulfide bond between C2 and C10. Unlabeled linear LyP-1 peptide (H-CGNKRTRGC-OH) (synthesized by AnaSpec) was used for competition experiment. The labeled peptide was used at a final concentration of 0.5 nM. Fluorescence polarization assay was performed at final buffer concentrations of 10 mM HEPES (pH 7.5), 150 mM NaCl, 20 mM MgCl_2_, 5 mM EDTA, 2% Glycerol, 10% DMSO. Recombinant human p32 (OriGene) was diluted in PBS and incubated with the peptide in the dark for 2 h with shaking. Controls for FP: (1) Labeled peptide alone without any protein and (2) Labeled peptide + p32 but without any inhibitor (DMSO only control). Fluorescence Polarization was measured with POLARstar Omega plate reader Ex485 Em520 (number of flashes 50) (Gain adjustment was on the no protein wells set to 75 mFP).

### Statistical analysis

Data are expressed as means ± standard deviations (SD) for all experiments. GraphPad Prism was used for statistical calculations and for graph generation.

## Results

### Pharmacophore modeling

**Fig. 1:**
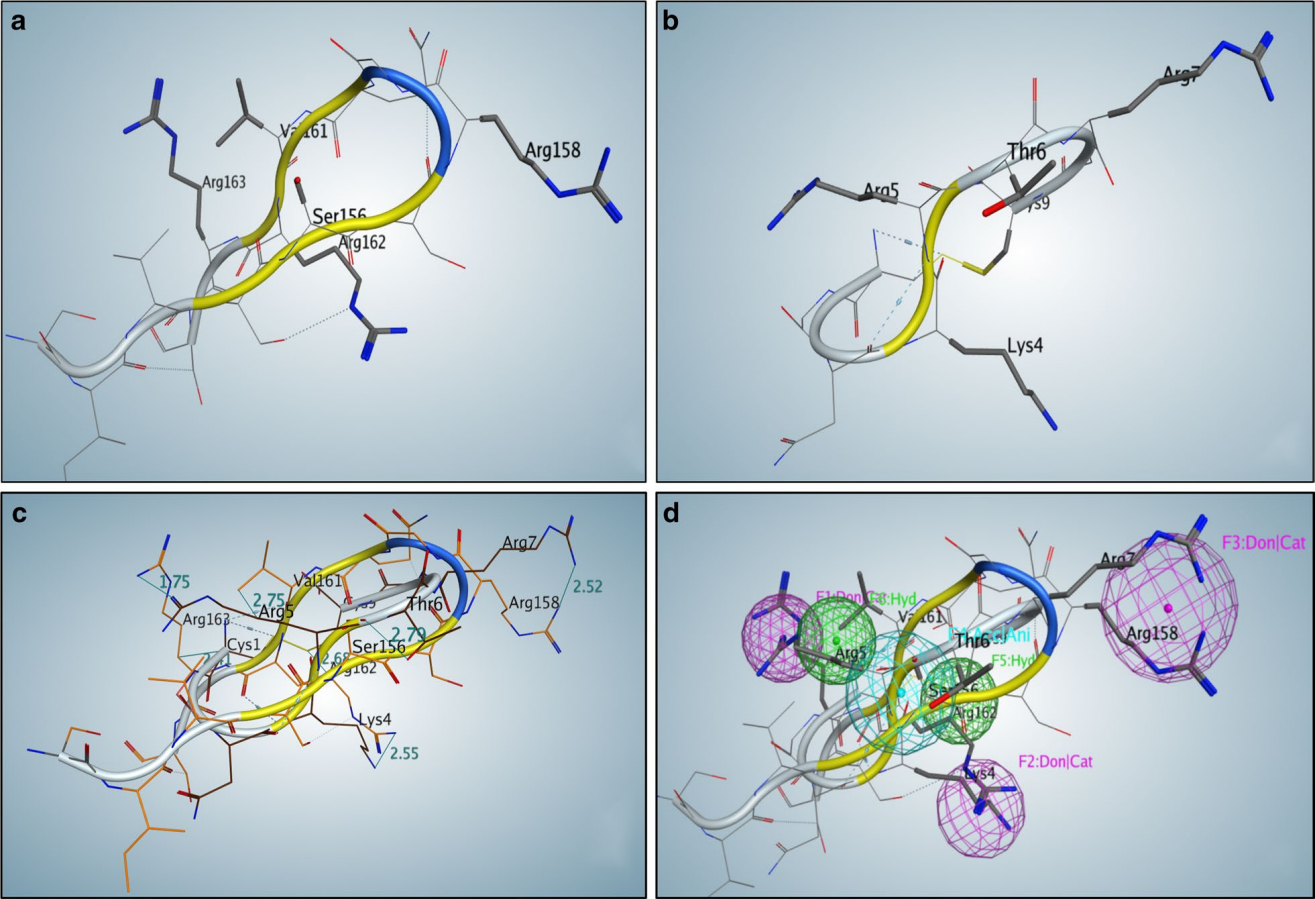
Pharmacophore model for p32. **a** 3D structure of the peptide-SIVSSSRGQVRRS from the p32-binding part of protein C1q. This structure is extracted from the known PDB structure of C1q. **b** 3D structure of LyP-1 cyclic peptide (CGNKRTRGC) modeled with the program InsightII (Accelrys, San Diego). **c** Superposition of above mentioned tertiary structures. **d** Assignment of six-feature pharmacophore centers. Both peptides have superimposable main features: 3 Donor|Cationic centers (violet: F1, F2, and F3), 2 Hydrophobic centers (green: F5 and F6), and 1 Acceptor|Anionic center (cyan: F4)

We developed a 3D configuration of the peptide LyP-1. To develop a pharmacophore model of LyP-1, we need to find its possible binding site to p32 and develop a pharmacophore. Since the crystal structure of Lyp-1 has not been solved, we built a model based on its nine-residue sequence, and later used its similarity to a fragment of C1q, which binds to p32. The P32 crystal structure (PDB ID: 1p32) has gaps in its chains. To make docking more accurate, we developed loops to fill these gaps, using p32 sequence and MOE Loop Modeler module. Docking with C1q showed a binding fragment of C1q containing 13 residues (152–164: SIVSSSRGQVRRS) that was separated (Fig. 1a). The binding residues of C1q are Ser156, Arg158, Val161, Arg162, and Arg163. Superposition with a built model of LyP-1 (1–9: CGNKRTRGC; Fig. 1b) showed functional similarity: hydrophilic Ser156–Thr6 2.79 Å, positive Arg158–Arg7 2.52 Å, hydrophobic Val161–hydrophobic part of Arg5 2.75 Å, positive Arg162–Lys4 2.55 Å, hydrophobic part of Arg162–Cys9 2.68 Å, positive Arg163–Arg5 1.75 Å, and hydrophobic part of Arg163–Cys1 2.41 Å (Fig. 1c).

We previously reported a pharmacophore modeling strategy for identifying compounds that could perturb the dimerization of the transcription factor Olig2 with its binding partner E47 [27]. The 3D configuration of positively charged residues of C1q (Arg158, Arg161, and Arg162) is very close to such configuration of positively charged residues of LyP-1 (Arg5, Lys4, and Arg7) in the superimposed structures (Fig. 1c). The consensus 3D-pharmacophore hypothesis would have a significant possibility to define the pharmacophore features as shown in Fig. 1d, and we hypothesized that a small molecule able to fit such pharmacophore would bind to the p32 binding site as the C1q and LyP-1, thereby inhibiting its function. A six-feature pharmacophore was developed with the MOE2015 Pharmacophore Query Editor. Pharmacophore centers were based on superimposed C1q–LyP-1 residues: F1:Donor/Cationic Arg158–Arg7; F2:Donor/Cationic: Arg162–Lys4; F3:Donor/Cationic: Arg163–Arg5; F4:Acceptor/Anionic: Ser156–Thr6; F5:Hydrophobic: hydrophobic part of Arg162–Cys9; and F6:Hydrophobic: Val161–hydrophobic part of Arg5. This six-feature pharmacophore model was used as the benchmark for a virtual screen of 3D conformational databases derived from the NCI Open Database (release 4, accessed June 2012) of 265,242 compounds. The search with six-of-six-feature pharmacophore returned 30 compounds. Of these, five had logBB values in the range − 0.3 to − 1.5; as this is indicative of an ability to penetrate the blood–brain barrier, only these compounds were included in future analyses. The five-of-six-feature pharmacophore run returned 3423 compounds, from which 109 were selected using a set of criteria including RMSD to the pharmacophore model, energies of conformation, and a set of pharmacophore-based descriptors.

To reduce experimental work, we clustered 109 resulting compounds with MOE Compute/Fingerprint/Clusters module, using the GpiDAPH3 fingerprint and similarity—overlap parameter *SO* = 40%. The results yielded 10 clusters with at least two members [one cluster (1A) with 63 members, one (1B) with eight members, four with three members, and five with two members], and 16 clusters with one member. Cluster 1B was the best: six of eight compounds have a property to penetrate the blood–brain barrier (range of logBB = + 0.35 to + 0.65), and in the cluster 1C all four compounds (range logBB = − 0.31 to − 0.83). Since cluster 1A was too general, we performed a second clustering run using the GpiDAPH2 fingerprint and an *SO* of 45%. As a result, we obtained 15 clusters, 1 (2A) cluster with 33 members, 1 cluster (2B) with 11 members, 1 cluster (2C) with 5 members, 2 clusters with 2 members, and 10 clusters with 1 member. The compounds in cluster 2B belong to the same family and their logBB exceed − 1.6. The logBB range of compounds in cluster 2B is − 0.76 to − 1.41 and in cluster 2C is + 1.59 to − 1.41. The third clustering run included the cluster 2A and was provided with GpiDAPH3 fingerprint and an SO of 53%. This yielded one cluster (3A) with eleven compounds, one cluster (3B) with two compounds, and 10 clusters with one compound. A list of 19 compounds from clusters 1B, 2C, and 3A was selected.

### Primary screening assay of potential p32 inhibitors

**Fig. 2:**
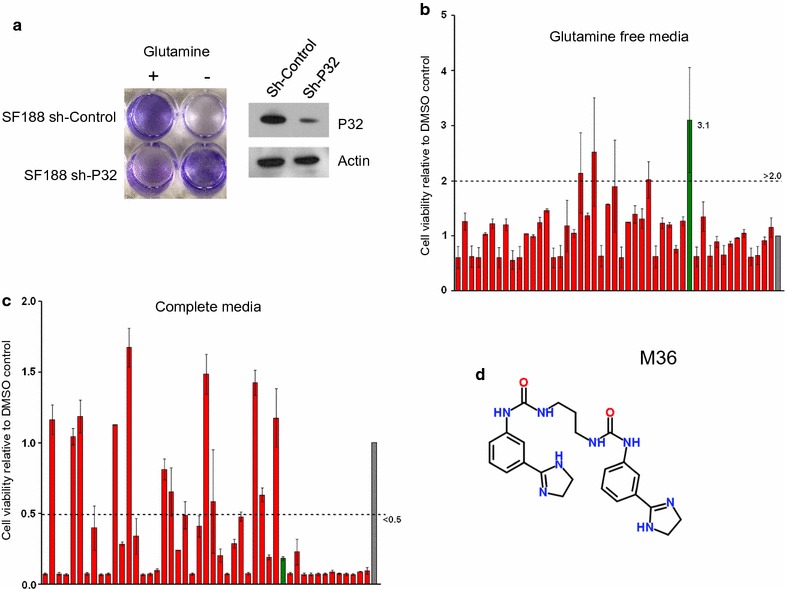
Primary assay screening p32 inhibitors for ability to rescue cells in glutamine free media. **a** Stable SF188 shControl cells or SF188 cells with genetic knockdown of p32 (shP32) were grown in glutamine free media for 3 days then allowed to recover 1 day in glutamine containing media. Cells were stained with crystal violet. **b** Potential p32 inhibitors were tested at 50 µM with SF188 glioma cells incubated in glutamine free DMEM (+ 10% dialyzed FBS), or **c** in complete media [DMEM with glutamine (+ 10% dialyzed FBS)], and cell viability was determined by Alamar Blue assay after 4 days. P32 inhibition promotes cell growth in glutamine free media and inhibits growth in complete media. Gray bar is DMSO control; green bar shows potential p32 inhibitor compound (M36). **d** Structure of small molecule M36

Myc-overexpressing cells have been shown to be addicted to glutamine [28–31]. We previously reported p32 is upregulated by Myc and has a role in regulating glutamine addiction [1]. P32 knockdown in glutamine addicted SF188 glioma cells can rescue the cells from death when grown in glutamine free media (Fig. 2a). Loss of p32 in these cells promotes a change from glutamine addiction to a dependence on glucose as a carbon source [1]. We established a screening assay to test compounds identified by virtual screening for rescue of p32-expressing glutamine addicted SF188 cells in glutamine free media. Testing the compounds at 50 µM identified several that could rescue cell death in glutamine free media and inhibit proliferation in media supplemented with glutamine (complete media) (Fig. 2b, c). One compound in particular (M36) was effective at rescuing cells from glutamine dependence and was an effective inhibitor of cell proliferation in complete media (Fig. 2d).

### Direct binding of small molecule M36 to p32

**Fig. 3:**
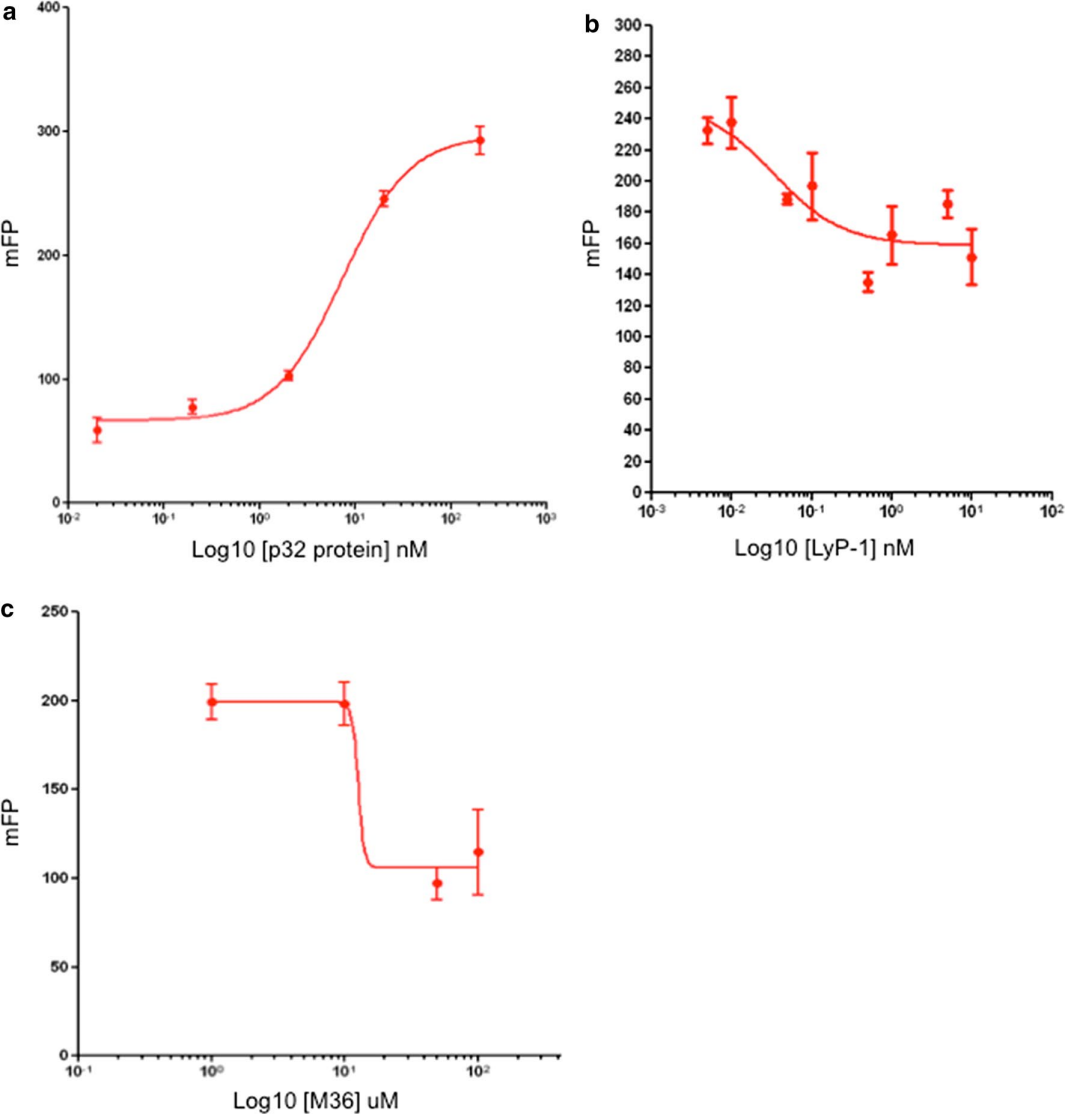
Fluorescence polarization assay demonstrating M36 binding to p32. **a** 5-FAM labeled cyclic LyP-1 peptide was incubated with increasing concentrations of recombinant p32 for 2 h. Increase in fluorescence polarization (mFP) indicates binding to the peptide. **b** 100 nM p32 was pre-incubated 1 h with increasing concentrations of unlabelled LyP-1 peptide in a competition assay before incubation with 0.5 nM labeled cyclic LyP-1 peptide. **c** 100 nM p32 was incubated with increasing concentrations of M36 for 30 min and then incubated with 5-FAM labeled cyclic LyP-1 peptide for 2 h. Direct binding of the hit compound M36 to p32 inhibits peptide binding resulting in decreased fluorescence polarization (mFP). Error bars represent standard deviation

In order to confirm the hypothesis of the pharmacophore model, we established a fluorescence polarization (FP) assay to validate M36 as a molecule interfering with p32 and LyP-1 peptide association. Cyclic LyP-1 peptide (G[C]GNKRTRG[C] [COOH]; disulfide bonded between C2 and C10) was fluorescently labeled at the amino terminus with 5-FAM (5-Carboxyfluorescein) and used in p32-binding assays. Binding of human recombinant p32 to the fluorescent-tagged LyP-1 peptide increases the size and enhances fluorescence polarization (Fig. 3a). To validate the assay we employed unlabeled LyP-1 peptide as a competitor for binding (Fig. 3b). We screened the inhibitor M36 in the FP assay and show it to be an effective inhibitor of p32 binding to labeled LyP-1 (Fig. 3c).

**Fig. 4:**
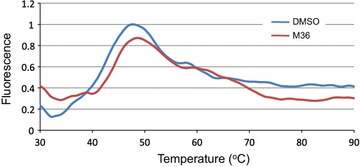
Protein thermal shift induced by M36 binding p32. Human recombinant protein p32 was incubated with 100 µM M36 or DMSO control and protein thermal shift determined with BioRad CFX96 real-time PCR

Although FP assay validates the pharmacophore model hypothesis, it does not demonstrate direct binding of M36 to p32. To demonstrate direct target binding we employed a protein thermal shift assay. Recombinant p32 was incubated with 100 µM M36 and protein thermal shift was assessed. P32 protein stability was altered by M36 as observed by a shift in the melting curve of the protein resulting from a direct interaction (Fig. 4). The combination of these assays suggests that the compound M36 binds directly to p32 and can interfere with LyP-1 binding.

### Growth inhibition and altered metabolism induced by M36

**Fig. 5:**
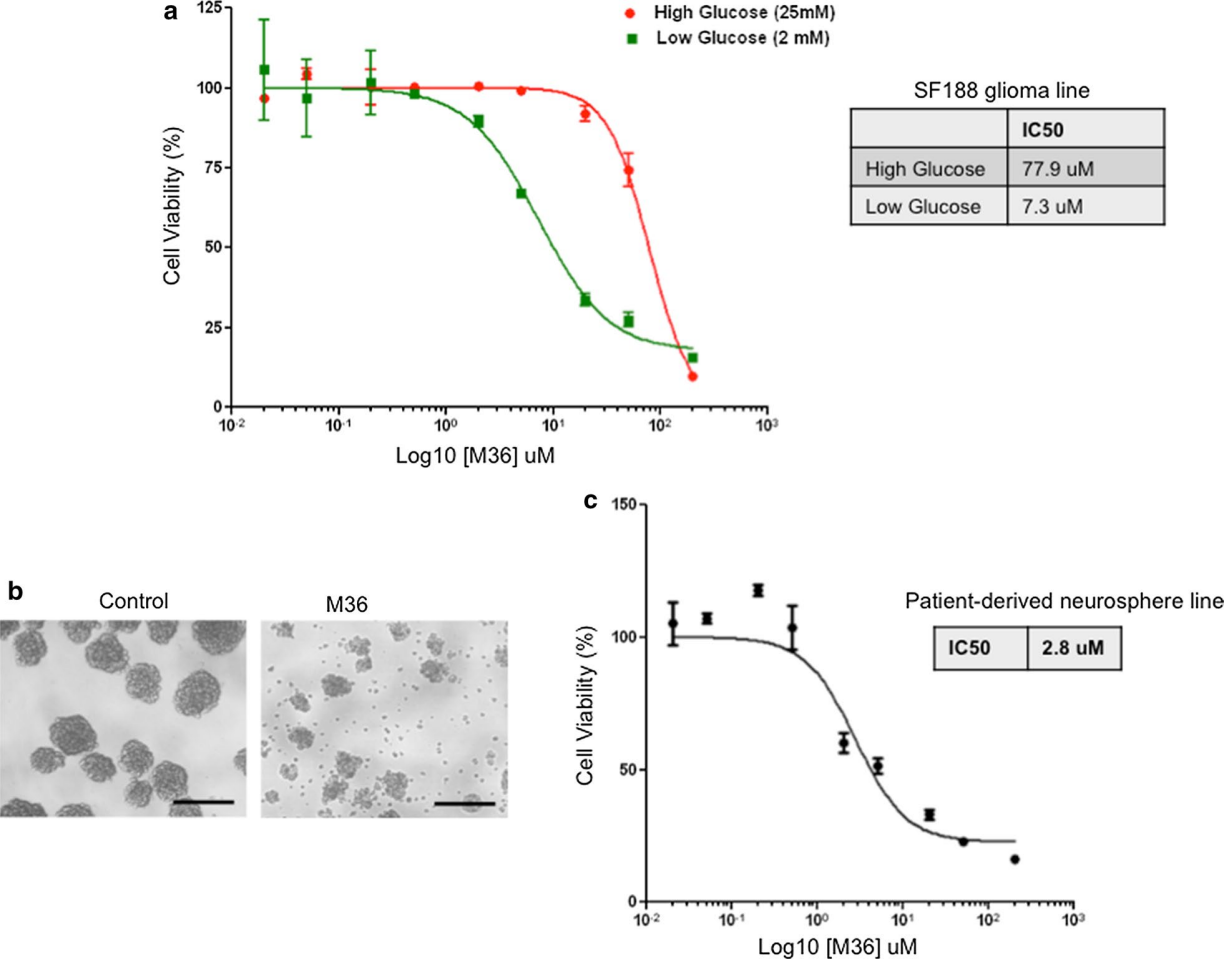
M36 inhibits glioma cell proliferation and is more potent under low glucose conditions. **a** SF188 glioma cells were incubated in high glucose (25 mM) or low glucose (2 mM) DMEM (+ 10% dialyzed FBS) and increasing concentrations of M36. After 4 days cell viability was determined by Alamar Blue assay and IC50 calculated using Graphpad Prism. **b** Patient-derived glioma neurosphere line GBM8 was incubated with DMSO or 20 µM M36 for 4 days. Cells were imaged showing decrease in size and number of neurospheres (scale bar = 200 μm). **c** GBM8 cells were incubated with increasing concentrations of M36 for 7 days. Cell viability was determined by Alamar Blue assay and IC50 calculated using Graphpad Prism

**Fig. 6:**
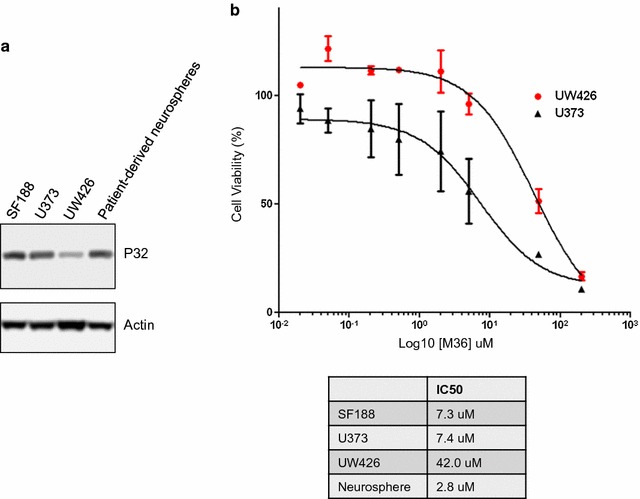
M36 selectively targets high p32 expressing cells. **a** Immunoblot for p32 for indicated cell lines. **b** U373 and UW426 cells were incubated in low glucose (2 mM) DMEM (+ 10% dialyzed FBS) with increasing concentrations of M36. Cell viability was determined by Alamar Blue assay and IC50 calculated using Graphpad Prism and was compared to the IC50 values for SF188 and patient-derived neurospheres

To test if the identified p32-binding compound M36 could inhibit p32 function we examined its ability to phenocopy a p32 genetic knockdown. Genetic knockdown of p32 in glioma cells can inhibit proliferation and sensitize the cells to glucose withdrawal [1]. We examined if M36 could inhibit glioma cell proliferation and if inhibition was more potent under low glucose conditions. Figure 5a shows M36 could inhibit glioma cell proliferation (IC50 of 77.9 µM in complete media (25 mM glucose)) and was much more potent under low glucose (2 mM) conditions with an IC50 of 7.3 µM. We previously reported p32 expression was upregulated in patient-derived neurospheres, and showed that these cells were highly sensitive to p32 knockdowns [1]. We treated these cells with M36 and observed a significant decrease in viability (Fig. 5b). M36 was a potent inhibitor of patient-derived neurospheres (IC50 2.8 µM) (Fig. 5c). To determine if M36 was selective for p32 expressing cells we examined its affect on cell viability of U373 glioma cell line (high p32 expressor) and UW426 medulloblastoma (low p32 expressor) (Fig. 6a). In 2 mM glucose the UW426 cell line was less sensitive to M36 than was U373 suggesting the compound was selective for p32 overexpressing cells (Fig. 6b).

**Fig. 7:**
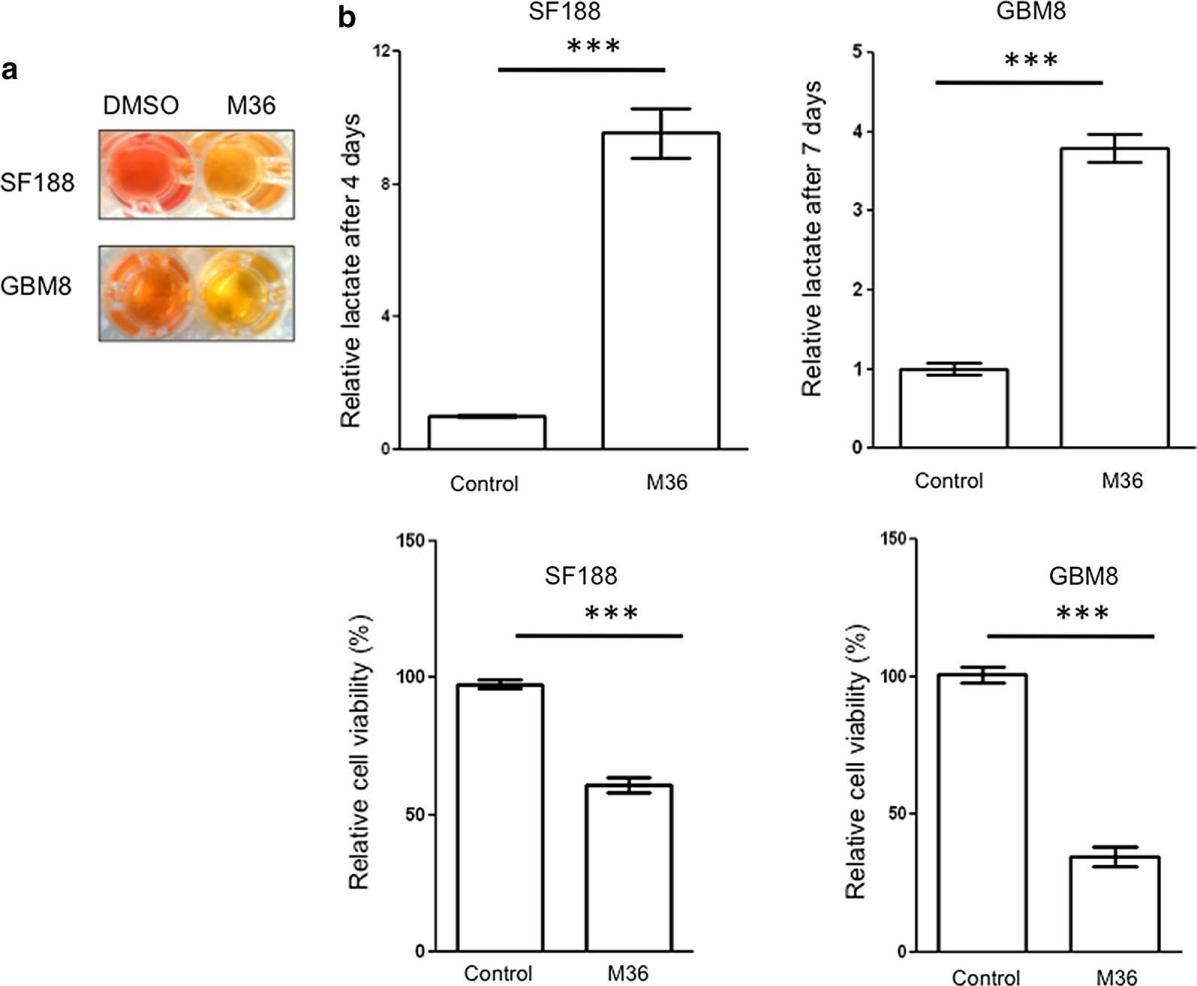
M36 promotes an increase in lactate production resulting in media acidification. **a** Culture media acidification for SF188 glioma cells and patient-derived GBM8 cells treated with 50 µM M36 for 4 and 7 days, respectively. **b** Lactate levels within the culture media were determined and relative lactate levels are shown after normalizing for cell number. The affect of M36 on cell proliferation is shown as relative cell viability (%). Bars represent mean ± standard deviation for 3 independent experiments (***p < 0.001)

Loss of p32 triggers a switch from mitochondrial OxPhos to aerobic glycolysis resulting in an increase in glucose consumption and lactate production. Treating glioma cells with M36 resulted in acidification of the culture media (color change from red to yellow) (Fig. 7a). This suggests an increase in lactate production and secretion as reported for p32 genetic knockdown [1, 8]. We assessed M36 for its ability to promote lactate production and secretion by examining if there was increased lactate in the culture media of treated cells. Figure 7b shows a significant amount of lactate within the culture media of treated SF188 and patient-derived GBM8 cells. Increased lactate within the media suggests M36 promoted a switch from mitochondrial OxPhos to aerobic glycolysis similar to p32 knockdown (Fig. 7).

## Discussion

The mitochondrial protein p32 is a validated cancer target overexpressed in glioblastoma and several other cancers [23]. Genetic knockdown of p32 limits cell proliferation in vitro and tumor growth in vivo. Therapeutic targeting p32 with monoclonal antibodies or the p32 homing peptide LyP-1 can limit tumor growth in vivo [18–20, 22]. However, these strategies only target surface p32 and may not target mitochondrial localized p32. In addition, antibodies and peptides may not readily cross the blood–brain barrier and may not be effective against central nervous system cancers. Our study was specifically designed to identify a small molecule inhibitor of mitochondrial p32 function that could be developed as an anti-glioblastoma therapeutic. A pharmacophore model of C1q and of LyP-1 tumor homing peptide showed significant similarity and could be superimposed. The consensus 3D pharmacophore identified compounds able to engage all proposed pharmacophores features. This strategy identifies molecules expected to interfere with both C1q and LyP-1 association with p32.

We previously reported that mitochondrial p32 has a role in metabolism of Myc overexpressing cells and showed loss of p32 attenuates Myc-mediated glutamine addiction [1]. Within the mitochondria it was proposed p32 functions to maintain mitochondrial integrity by regulating the expression of mitochondrial-DNA-encoded respiratory chain complexes [8, 10]. Mitochondria are essential for cancer cell survival acting as metabolic sensors that can trigger cell death through cytochrome *c* release [32]. Recent reports have provided genetic and pharmacological evidence that mitochondrial metabolism is essential for tumor growth [8, 33, 34]. Mitochondrial inhibitors such as metformin, tigecycline, and VLX600 have demonstrated success in pre-clinical models of cancer [34–36]. Accumulating research thus demonstrates that targeting mitochondria metabolism of tumors can be an effective therapeutic strategy [35, 36]. In addition, recent studies have demonstrated glioma initiating stem-like cells (GSCs) rely heavily on mitochondrial OxPhos for bioenergetics and survival [37]. GSCs can alter metabolism to support either a quiescent phenotype or rapidly proliferating phenotype depending on available nutrients and microenvironment. The design or identification of compounds that inhibit such metabolic plasticity offers a therapeutic window for sensitizing cancer cells to conventional therapy [36, 38, 39]. Directed targeting of GSC mitochondria in combination with conventional therapy (radiation and chemotherapy) may help eradicate GSCs and limit tumor recurrence. M36 represents a new class of compounds targeting an important mitochondrial regulatory protein. It is a potent inhibitor of patient-derived GSC line GBM8, and limits metabolic plasticity by targeting mitochondrial metabolism, forcing cells to use aerobic glycolysis for bioenergetics. This sensitizes the cells to depletion of glucose and other nutrients, and will likely also render them susceptible to glycolytic inhibitors. Future studies will examine whether M36 can synergize with chemotherapy or radiation to inhibit tumor growth, and whether the combination of a glycolysis inhibitor with p32 inhibitor can block tumor growth.

M36 effectively antagonized the interaction between p32 and LyP-1 peptide. In addition, direct binding of M36 to p32 was observed by thermal shift thus validating the pharmacophore model. LyP-1 can direct nanoparticles to p32-expressing tumors [20]. Similarly, M36 has potential as a drug delivery agent based on its ability to bind p32, which becomes overexpressed on the surface of cancer cells [12] as has been reported for other p32-directed delivery compounds [40]. Thus M36 has potential not just as an inhibitor of mitochondrial function in cancer cells overexpressing p32, but also as a nanoparticle homing agent directed to tumors that overexpress p32 at the cell surface. Future experiments will determine whether M36 has efficacy at limiting tumor growth in vivo and whether it binds to cell surface expressed p32.

## Conclusions

Pharmacophore modeling of p32 association with the tumor homing peptide LyP-1 and C1q identifies potential p32 binding molecules. One of these molecules is shown to inhibit p32 function and inhibit proliferation of glioma lines overexpressing p32. In conclusion, the validation of this screening strategy offers potential to identify potent and selective inhibitors of mitochondrial p32 amenable for further drug development.
